# Suppressor of cytokine signaling 2 (*Socs2*) deletion protects bone health of mice with DSS-induced inflammatory bowel disease

**DOI:** 10.1242/dmm.028456

**Published:** 2018-01-01

**Authors:** Ross Dobie, Vicky E. MacRae, Chloe Pass, Elspeth M. Milne, S. Faisal Ahmed, Colin Farquharson

**Affiliations:** 1 Division of Developmental Biology, The Roslin Institute and Royal (Dick) School of Veterinary Studies, University of Edinburgh, Easter Bush, Roslin, Midlothian, Edinburgh EH25 9RG, UK; 2 School of Medicine, University of Glasgow, Royal Hospital for Children, Govan Road, Glasgow G51 4TF, UK

**Keywords:** SOCS2, Inflammatory bowel disease, IBD, Growth hormone, Bone

## Abstract

Individuals with inflammatory bowel disease (IBD) often present with poor bone health. The development of targeted therapies for this bone loss requires a fuller understanding of the underlying cellular mechanisms. Although bone loss in IBD is multifactorial, the altered sensitivity and secretion of growth hormone (GH) and insulin-like growth factor-1 (IGF-1) in IBD is understood to be a critical contributing mechanism. The expression of suppressor of cytokine signaling 2 (SOCS2), a well-established negative regulator of GH signaling, is stimulated by proinflammatory cytokines. Therefore, it is likely that SOCS2 expression represents a critical mediator through which proinflammatory cytokines inhibit GH/IGF-1 signaling and decrease bone quality in IBD. Using the dextran sodium sulfate (DSS) model of colitis, we reveal that endogenously elevated GH function in the *Socs2^−/−^* mouse protects the skeleton from osteopenia. Micro-computed tomography assessment of DSS-treated wild-type (WT) mice revealed a worsened trabecular architecture compared to control mice. Specifically, DSS-treated WT mice had significantly decreased bone volume, trabecular thickness and trabecular number, and a resulting increase in trabecular separation. In comparison, the trabecular bone of *Socs2*-deficient mice was partially protected from the adverse effects of DSS. The reduction in a number of parameters, including bone volume, was less, and no changes were observed in trabecular thickness or separation. This protected phenotype was unlikely to be a consequence of improved mucosal health in the DSS-treated *Socs2*^−/−^ mice but rather a result of unregulated GH signaling directly on bone. These studies indicate that the absence of SOCS2 is protective against bone loss typical of IBD. This study also provides an improved understanding of the relative effects of GH/IGF-1 signaling on bone health in experimental colitis, information that is essential before these drugs are explored as bone protective agents in children and adults with IBD.

## Abstract (precis)

**Summary:** Using a mouse model of inflammatory bowel disease, this article provides an improved understanding of the relative effects of GH/IGF-1 on bone health in experimental colitis.

## INTRODUCTION

Inflammatory bowel disease (IBD), which includes the chronic intestinal disorders Crohn's disease (CD) and ulcerative colitis (UC), is considered to result from an inappropriate inflammatory response to intestinal microbes in genetically susceptible hosts (Abraham and Cho, 2009). They are life-long conditions with a prevalence of 5 per 1000 people and annual healthcare costs exceeding US$1.7 billion in the United States (Sandler et al., 2002; Lakatos, 2006). In addition to the well-recognized gut inflammation associated with IBD, both children and adults have poor bone health and are at increased risk of fractures that cannot solely be attributed to exogenous glucocorticoid exposure (Thearle et al., 2000; van Staa et al., 2003; Wong et al., 2016). The relative risk of fracture in adults is 40% higher than normal (Compston et al., 1987) and a vertebral fracture may be present in 10% of affected people (Laakso et al., 2012; Wong et al., 2014). Recent studies in children have also shown that trabecular bone density, which was reduced at diagnosis, showed inadequate improvement despite control of the underlying inflammation (Dubner et al., 2009). These patients also have abnormal bone geometry, with thinner and smaller bones (Dubner et al., 2009; Tsampalieros et al., 2013). Furthermore, peak bone mass is compromised in those with childhood-onset IBD, despite adequate control of disease and progression through puberty (Laakso et al., 2014).

The etiology of bone loss in IBD is multifactorial, with risk factors including steroid medication, poor nutrient intake/absorption and the underlying inflammatory state (Bernstein and Leslie, 2003). Central to the inflammatory response in IBD is the overproduction by T cells and macrophages of various proinflammatory cytokines, such as interleukin (IL)-1, -2, -6 and -8, and tumor necrosis factor (TNF)-α (Ali et al., 2009). IL-6 has been identified as the predominant cytokine mediating the bone abnormalities, and genetic variations in *IL-6* correlate well with the clinical course of IBD and the extent of bone loss (Schulte et al., 2000). Although proinflammatory cytokines are known to promote bone loss directly, they also lead to altered sensitivity and secretion of growth hormone (GH) and insulin-like growth factor-1 (IGF-1) in IBD, which may be another critical mechanism leading to osteoporosis (Wong et al., 2010).

GH and IGF-1 are recognized stimulators of bone mass (Giustina et al., 2008). Transgenic mice overexpressing *Igf1* in osteoblasts exhibit increased trabecular bone, whereas *Igf1*-null mutants exhibit reduced cortical bone (Liu et al., 1993; Zhao et al., 2000). GH receptor (*GHR*)^−/−^ mice have reduced bone turnover, cross-sectional cortical bone area and cortical growth, whereas GH overexpression results in increased bone cortical area (Sjogren et al., 2000; Sims et al., 2000; Eckstein et al., 2004). Skeletal manifestations are also observed in humans with GH deficiency, who present with low-bone-turnover osteoporosis (Doga et al., 2005), which can be ameliorated by recombinant human (rh)GH replacement therapy (Hansen et al., 1996). The therapeutic benefits of rhGH administration on bone health of IBD patients remain unknown. Nevertheless, given that both rhGH and rhIGF-1 are available as therapeutic drugs, there is potential for these anabolic drugs to improve bone health in people with IBD.

Suppressor of cytokine signaling 2 (SOCS2) regulates GH signaling during normal growth and development. This physiological role is not restricted to bone. This function is most clearly observed in the *Socs2* knockout (KO) mouse, in which a number of organs are increased in size and these include liver, heart, lungs and bladder (Metcalf et al., 2000). In another study, SOCS2 has been shown to limit intestinal growth (Michaylira et al., 2006). SOCS2 is also induced by a subset of proinflammatory cytokines [e.g. IL-6, IL-1β and TNF-α, which are elevated in IBD and mediate the inflammatory response (Starr et al., 1997; Greenhalgh and Hilton, 2001; Rico-Bautista et al., 2006; Sanchez-Muñoz et al., 2008; MacRae et al., 2009)]. However, this protection against inflammation may come at the expense of poor bone health through the induction of SOCS2 and the inhibition of GH signaling (Denson et al., 2003; Shi et al., 2004).

SOCS2 is expressed by many cells, including osteoblasts, and inhibits GH signaling via inhibition of the JAK/STAT intracellular signaling pathway (Greenhalgh et al., 2002; Flores-Morales et al., 2006; Michaylira et al., 2006; Pass et al., 2012; Dobie et al., 2014). *Socs2-*deficient mice have unrepressed GH signaling and present with an overgrowth and high-bone-mass phenotype (Metcalf et al., 2000; MacRae et al., 2009; Dobie et al., 2014). These data emphasize the critical role of SOCS2 in controlling the anabolic effects of GH on bone. Therefore, it is possible that increased osteoblast SOCS2 expression represents a critical mediator through which proinflammatory cytokines inhibit GH/IGF-1 signaling and decrease bone mass and quality in patients with IBD.

To directly examine this, we studied bones from a mouse model of IBD using dextran sodium sulfate (DSS)-induced colitis and investigated the potential of ablating the expression of *Socs2* to endogenously elevate GH and IGF-1 function, and correct the bone loss observed in murine colitis. This study is the first in an IBD animal model to investigate whether GH and IGF-1 have bone protective effects. Furthermore, data from this innovative approach will help inform the design of novel therapies that are directed specifically at the mechanism of insult that leads to poor bone health in children and adults with IBD.

## RESULTS

### Effect of DSS treatment on body weight of WT and *Socs2*^−/−^ mice

**Fig. 1.**
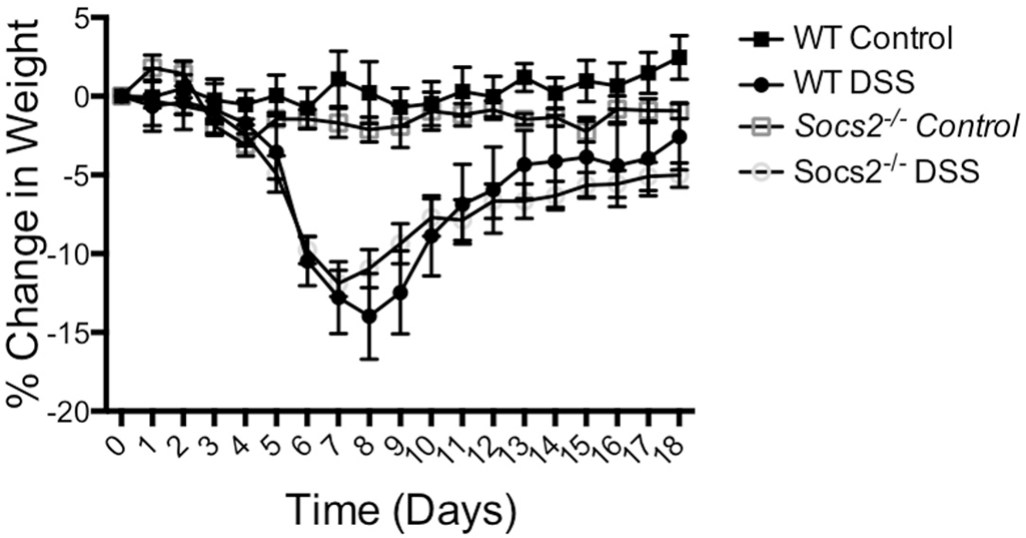
**Body-weight changes of WT and *Socs2^−/−^* mice treated with DSS followed by a recovery period.** Percentage change in body weight of WT and *Socs2^−/−^* mice treated with 3% DSS for 4 days. Data are presented as means±s.e.m. (*n*=6).

To investigate the effects of DSS-induced colitis on bone development and the possible role of SOCS2 in mediating bone loss, WT and *Socs2^−/−^* mice were treated with 3% DSS for 4 days (Fig. 1). The dose and duration of DSS treatment was based on results from a dosing experiment where mice were given DSS at varying concentrations for 4-5 days (Fig. S1). During the DSS treatment period (0-4 days), no weight loss was observed in WT or *Socs2^−/−^* mice (Fig. 1). Independent of genotype, mice exhibited a rapid weight loss from day 4 (Fig. 1). There was no significant difference in maximum weight loss observed between WT (15%) and *Socs2^−/−^* (13%) mice (Fig. S1B). Following the period of weight loss, all DSS-treated mice continued to gain weight to the end of the study (day 18) (Fig. 1). Pair-fed control mice did not show any significant alterations in body weight throughout the experiment (Fig. 1). During the period of DSS treatment, water intake was similar in WT and *Socs2^−/−^* mice. Also, food intake during DSS intake and the periods of weight loss and recovery were similar (data not shown).

In terms of weight loss, WT mice showed high individual variation in susceptibility to DSS (Fig. S1C). Maximum body-weight loss ranged from 6 to 21% in WT mice treated with 3% DSS. In contrast, body-weight loss observed in *Socs2^−/−^* mice treated with 3% DSS was more uniform, ranging from 11 to 15% before recovering (Fig. S1C).

### Effect of DSS treatment on longitudinal growth of WT and *Socs2*^−/−^ mice

*Socs2^−/−^* mice are characterized by their overgrowth phenotype (Greenhalgh et al., 2002). In agreement with this, the untreated *Socs2^−/−^* mice at the end of this study (day 18) were 39% (*P*<0.001) heavier than untreated WT mice (Table 1). Also, tibia (6%; *P*<0.01) and femur (8%; *P*<0.01) length as well as nose-to-rump length (13%; *P*<0.001) were all greater in untreated *Socs2^−/−^* mice compared to WT mice (Table 1). DSS treatment had no effect on the body weight of WT and *Socs2^−/−^* mice at the end of the study (Table 1). Similarly, DSS treatment did not result in altered nose-to-rump length, tibia length or femur length of WT or *Socs2^−/−^* mice (Table 1).

**Table 1.**
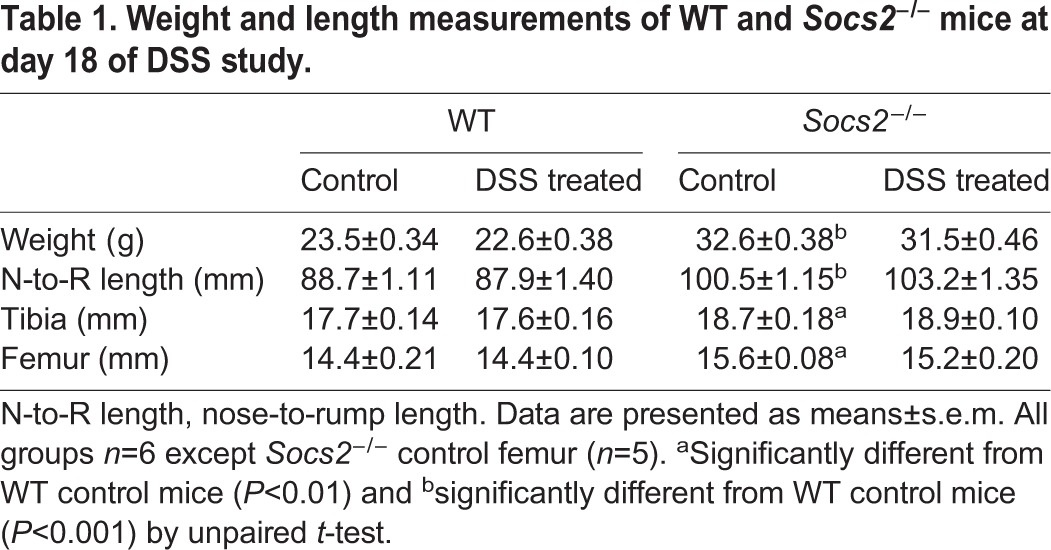
**Weight and length measurements of WT and *Socs2^−/−^* mice at day 18 of DSS study.**

### Colon pathology of DSS-treated mice

**Fig. 2.**
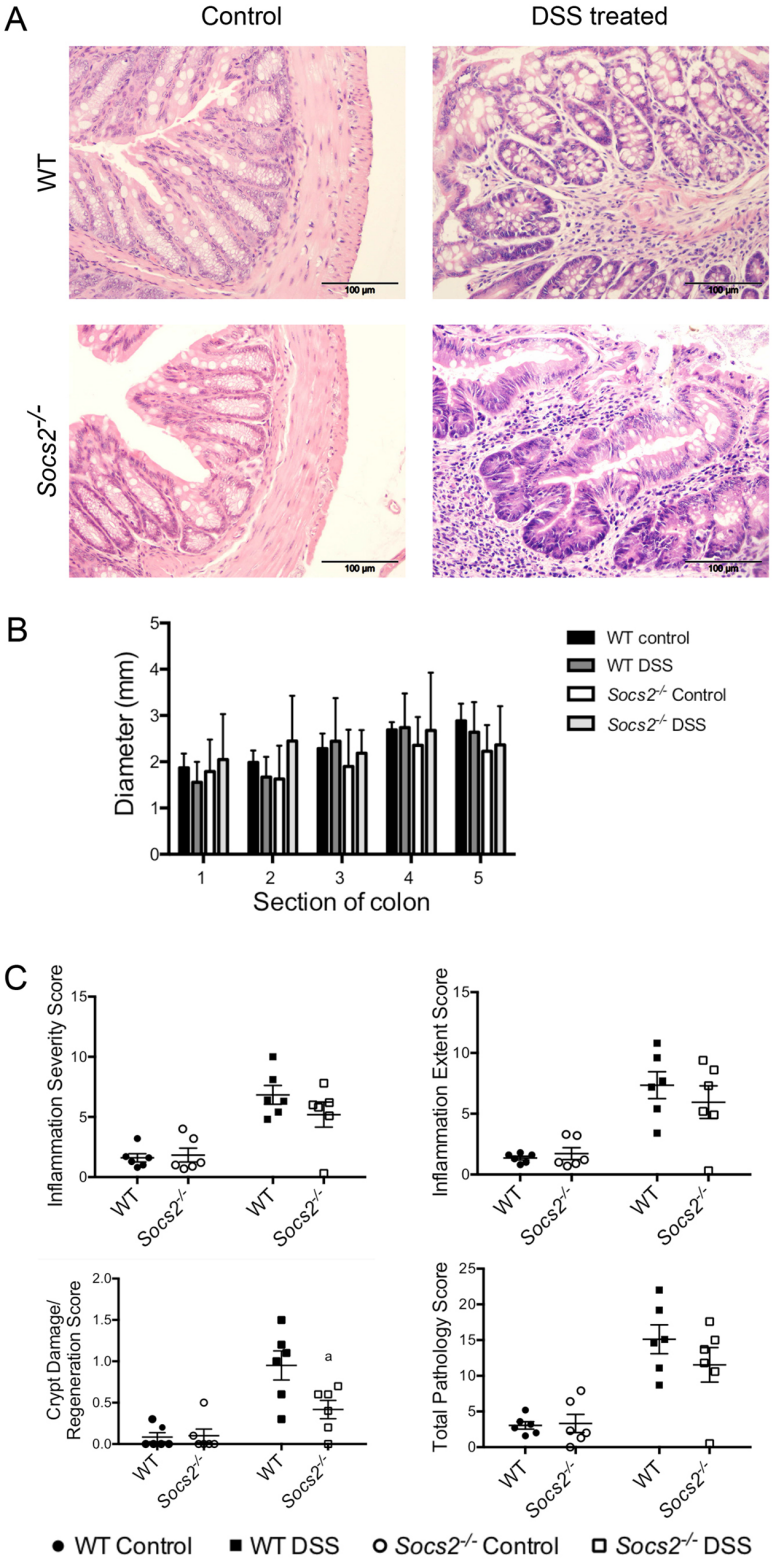
**Colon pathology of DSS-treated WT and *Socs2^−/−^* mice.** (A) Representative H&E-stained sections of colon from control and DSS-treated WT and *Socs2^−/−^* mice. (B) Diameter of DSS-treated and control WT and *Socs2^−/−^* colons. Numbers 1-5 represent different segments along the colon. (C) Histological scoring of DSS-treated and control WT and *Socs2^−/−^* colons. Data are presented as means±s.e.m. (*n*=6). ^a^Significantly different from DSS-treated WT mice (*P*<0.05) by unpaired *t*-test or nonparametric test (Mann–Whitney).

To assess the effects of DSS on mucosal integrity, detailed histological analysis was performed on the colon from control and DSS-treated WT and *Socs2^−/−^* mice. Histology scores were minimal in the non-DSS-treated (control) mice and, furthermore, there were no notable differences in colon diameter, morphological differences or differences in histological scoring between the non-DSS-treated (control) WT and *Socs2^−/−^* mice (Fig. 2A-C). This infers that the ablation of *Socs2* alone had no obvious effects on colon morphology. In contrast, histological analysis of the colon from DSS-treated WT and *Socs2^−/−^* mice revealed extensive levels of inflammation. DSS-treated mice were characterized as having signs of both acute and chronic inflammation throughout their colon (Fig. 2A). Signs of acute inflammation included infiltration of neutrophils into the lamina propria and submucosa (Fig. 2A), and epithelial degeneration (Fig. S2). In a number of sections there were also signs of crypt loss (Fig. S2). In addition to acute inflammation, there were also high levels of mononuclear leukocytes (macrophages, lymphocytes and plasma cells) (Fig. 2A and Fig. S2), and transmural inflammation (Fig. S2), which are recognized markers of chronic inflammation.

Scores for inflammation severity and extent of inflammation were significantly increased in DSS-treated mice (Fig. 2B). However, the histological mean scores for inflammation severity (WT: 6.8; *Socs2^−/−^*: 5.2) and extent of inflammation (WT: 7.4; *Socs2^−/−^*: 6.0) following DSS treatment were not significantly different between genotypes, nor was colon diameter (Fig. 2B,C). In contrast, there was a significant difference in crypt damage/regeneration mean score between DSS-treated WT and DSS-treated *Socs2^−/−^* mice (*P*<0.05) (Fig. 2B). This difference in crypt damage/regeneration between genotypes following DSS treatment was not sufficient, however, to alter total pathology scores, indicating that the absence of *Socs2* was unable to confer overall protection against DSS-induced gut inflammation (Fig. 2B). Although not part of the scoring system, the number of goblet cells in the mucosa of the colon was reduced in areas of loss of the epithelium as a result of the inflammatory response, and in areas of crypt damage and regeneration in DSS-treated WT and *Socs2*-deficient mice.

### Systemic IGF-1 levels in DSS-treated mice

GH resistance has been associated with IBD, and is characterized by a decrease in systemic IGF-1 levels (Ballinger et al., 2000; Katsanos et al., 2001). Analysis of serum from DSS-treated animals revealed that IGF-1 levels were similar in control and DSS-treated WT (WT control: 263.8±6.2 ng/ml; DSS-treated WT: 243.6±10.8 ng/ml) and *Socs2^−/−^* (*Socs2^−/−^* control: 264±9.8 ng/ml; DSS-treated *Socs2^−/−^*: 276.9±17.6 ng/ml) mice.

### Socs transcript levels in bones of DSS-treated mice

**Fig. 3.**
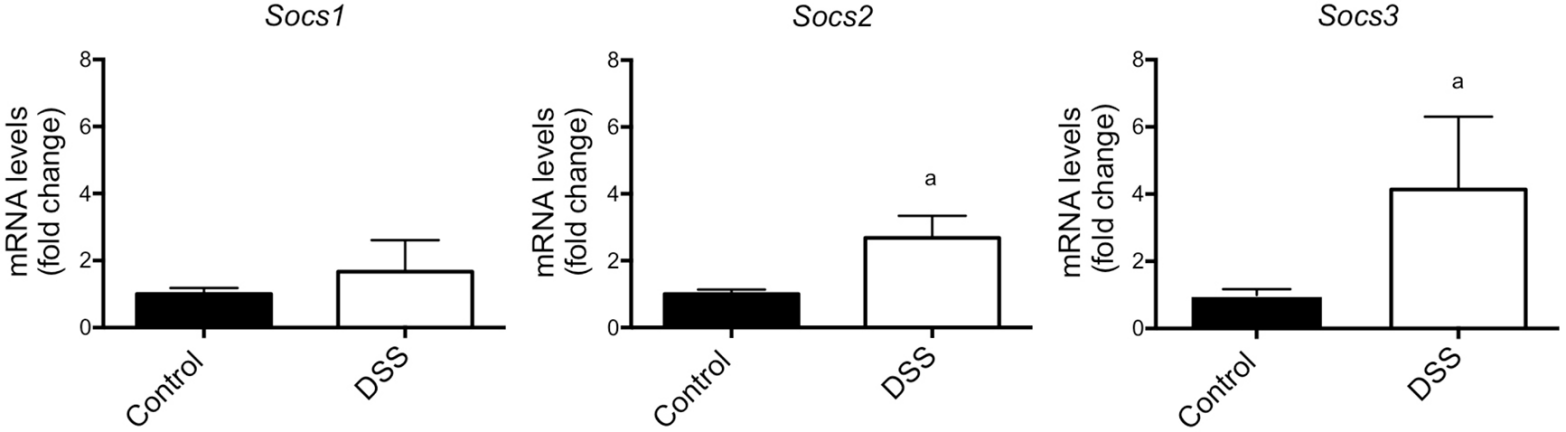
**Socs mRNA expression in bone from DSS-treated WT mice.** *Socs1*, *2* and *3* mRNA expression levels in tibia from DSS-treated WT mice compared to control mice at day 18 (endpoint) of the DSS study. Data are presented as means±s.e.m. Control group, *n*=6; DSS-treated group, *n*=4. ^a^Significantly different from control samples (*P*<0.05) by nonparametric test (Mann–Whitney).

*Socs1*, *2* and *3* transcript levels were measured in bone samples from control and DSS-treated WT mice. *Socs2* (2.7-fold; *P*<0.05) and *Socs3* (4.1-fold; *P*<0.05) levels were higher in DSS-treated mice, but no significant difference was observed in *Socs1* levels (Fig. 3).

### Bone phenotype of DSS-treated mice

**Fig. 4.**
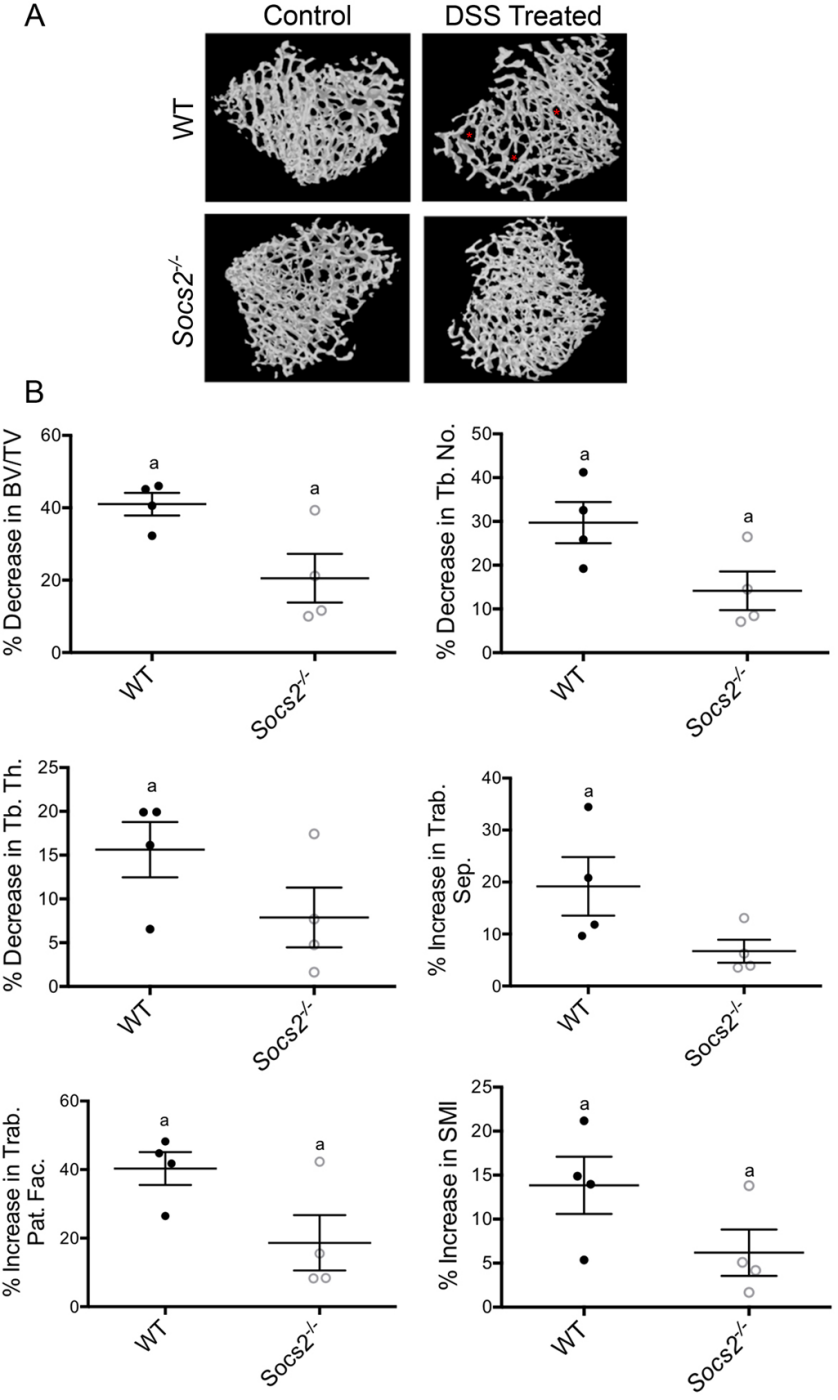
**Trabecular bone architecture of DSS-treated WT and *Socs2^−/−^* mice.** (A) Representative 3D μCT reconstructions showing a less-compact trabecular architecture resulting in a more porous structure (*) in DSS-treated WT mice compared to WT control mice. A similar alteration in bone architecture was not observed in DSS-treated *Socs2*-deficient mice. (B) Percentage change of trabecular parameters in DSS-treated mice relative to genotype-matched controls. Data are presented as means±s.e.m. (*n*=4). ^a^Significantly different from genotype-matched control (*P*<0.05) by nonparametric test (Mann–Whitney). BV/TV, bone volume/tissue volume; Tb., trabecular; Th., thickness; Pat. Fac., pattern factor; SMI, structural model index.

DSS-induced colitis has previously been shown to have detrimental effects on bone quality, in both juvenile (4-week-old) and young-adult (10-week-old) mice (Hamdani et al., 2008; Harris et al., 2009). In accordance with these studies, DSS-treated WT mice showed worsened trabecular architecture compared to control mice as demonstrated by micro-computed tomography (μCT) (Fig. 4). Specifically, DSS-treated WT mice had significantly decreased bone volume/tissue volume (BV/TV) (41%; *P*<0.05), trabecular thickness (16%; *P*<0.05) and trabecular number (30%; *P*<0.05), and a resulting increase in trabecular separation (19%; *P*<0.05) (Fig. 4). The increase in trabecular pattern factor (40%; *P*<0.05) and structural model index (14%; *P*<0.05) indicates a more disconnected ‘rod-like’ trabecular structure, which is associated with reduced trabecular microarchitecture quality (Hildebrand and Rüegsegger, 1997). In comparison, DSS-treated *Socs2^−/−^* mice showed a much less severe alteration in trabecular architecture compared to control mice (Fig. 4). Specifically, the reduction in BV/TV (21%; *P*<0.05) and trabecular number (14%; *P*<0.05), and increased trabecular pattern factor (19%; *P*<0.05) and structural model index (6%; *P*<0.05) were 50-55% less severe than that noted in DSS-treated WT mice (Fig. 4B). Furthermore, trabecular thickness and trabecular separation, in contrast to DSS-treated WT mice, were not significantly different in DSS-treated *Socs2*^−/−^ mice compared to controls (Fig. 4B). These striking data indicate that the level of bone loss in DSS colitis is influenced by *Socs2* expression and furthermore suggest that the absence of SOCS2 is partially protective against the bone loss that is typical of IBD.

Analysis of cortical bone in control and DSS-treated WT and *Socs2*-deficient mice revealed no change in any parameters following DSS treatment (Table 2). Nonetheless, the anabolic bone phenotype [characterized by increased cortical tissue area (*P*<0.05), bone area (*P*<0.05), cortical thickness (*P*<0.05) and marrow area (*P*<0.05)] was apparent in untreated *Socs2^−/−^* mice (Table 2).

**Table 2.**
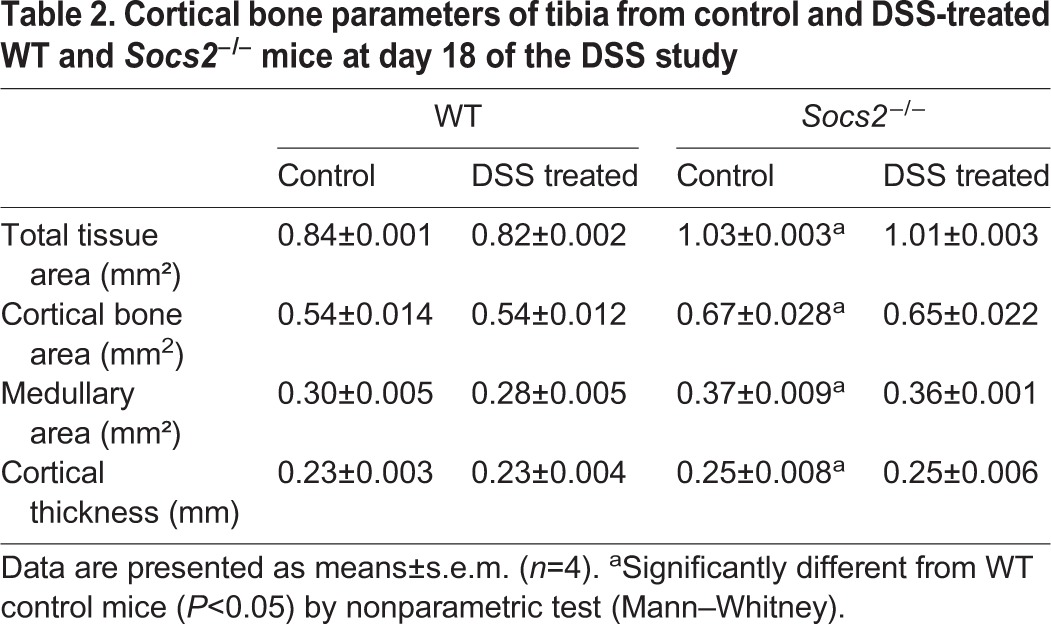
**Cortical bone parameters of tibia from control and DSS-treated WT and *Socs2^−/−^* mice at day 18 of the DSS study**

## DISCUSSION

Murine models of IBD have been used extensively to better understand disease etiology and therapeutic opportunities. In this study we used the DSS-induced colitis mouse model, which has been extensively validated by others to induce acute and chronic forms of IBD (Wirtz et al., 2007; De Roberts et al., 2011; Kanneganti et al., 2011). This model has also been used to study the link between IBD and the risk of colorectal cancer (Bollrath et al., 2009). The strength of this model is that histological changes seen can include a wide range of features that are associated with chronic forms of IBD in man (Perše and Cerar, 2012). DSS-induced colitis is the result of deterioration of the epithelial barrier through an increase in epithelial cell apoptosis and a decrease in proliferation (Araki et al., 2010). This deterioration allows for the influx of antigens and microorganisms, prompting increased expression of inflammatory mediators (e.g. TNF-α, IL-1β, IFN-γ, IL-10, IL-6 and IL-12) that drive the pathogenesis of DSS-induced colitis (Tlaskalová-Hogenová et al., 2005; Perše and Cerar, 2012).

DSS-induced acute colitis has previously been shown to result in reduced bone health in juvenile and young-adult mice. During active disease and early recovery in juvenile mice, both the trabecular and cortical bone compartments are adversely affected, whereas DSS-treated older mice exhibit reduced femoral bone mass and altered microarchitecture. No changes were observed in cortical bone indices (Hamdani et al., 2008; Harris et al., 2009).

In the present study, we found a severe trabecular bone loss phenotype in WT mice treated with DSS, but no changes in cortical bone parameters. These data are consistent with previous reports in similarly aged adult mice (Hamdani et al., 2008). The trabecular bone phenotype was characterized by lowered BV/TV, trabecular thickness and trabecular number, and by increased trabecular separation. Taken together, these studies suggest that there is an age-specific effect of DSS-induced IBD on bone loss in mice. During early puberty (3-5 weeks) there is extensive radial bone expansion and this may explain why the loss of cortical bone is restricted to juvenile mice (Callewaert et al., 2010). DSS treatment did not result in altered nose-to-rump length, tibia length or femur length of WT or *Socs2^−/−^* mice. This is in contrast to a previous study that reported decreased bone length and growth plate thickness and altered chondrocyte marker gene expression in young mice (4 weeks old) treated with DSS (Harris et al., 2009). Whilst bone length was fully restored at the end of the study these data are in accord with human studies that show IBD can stunt growth and diminish final height (Burnham et al., 2004; Sylvester et al., 2007). It is likely that we did not see a reduction in bone length in this study due to the older mice (8-9 weeks of age) being studied, by which time the rapid phase of linear bone growth was completed.

SOCS2 expression is induced by proinflammatory cytokines and is a recognized inhibitor of GH signaling (Denson et al., 2003). Furthermore, *Socs2*-deficient mice have uninhibited GH signaling, leading to increased body weight and a high bone mass phenotype (Metcalf et al., 2000; MacRae et al., 2009; Dobie et al., 2014). GH is a recognized stimulator of bone mass (Giustina et al., 2008). Global GH overexpression results in increased cortical cross-sectional area (Eckstein et al., 2004). Increased cortical area and bone strength is also observed in human GH transgenic mice, where expression of GH is directed specifically to osteoblasts, with minimal systemic overproduction (Tseng et al., 1996). Conversely, mice carrying mutations of the GH receptor have reduced femoral width and tibial cortical thickness, and an associated reduction in periosteal bone growth (Sims et al., 2000). Skeletal manifestations are also observed in humans with GH deficiency, who present with low bone turnover osteoporosis, leading to increased fracture risk (Doga et al., 2005). Using an osteoblast culture model, we have previously shown that the promotion of both STAT5 phosphorylation and its nuclear translocation by GH is enhanced in *Socs2-*deficient osteoblasts, whereas *Socs2* overexpression in osteoblasts blunts the effect of GH (Dobie et al., 2014). These observations stress the key role for SOCS2 in controlling GH's anabolic skeletal effects and are extended by data from this present study, which reveals that *Socs2* expression is higher in bone samples from DSS-treated WT mice compared with controls. This increased *Socs2* expression, a probable consequence of higher levels of circulating proinflammatory cytokines, is likely to repress GH signaling and contribute to the poor bone health noted in colitic (DSS-treated) WT mice.

In this study, we examined the hypothesis that elevated GH signaling in mice deficient in SOCS2 protein would offer protection against bone loss in a murine model of IBD. Such protection was observed in trabecular bone, where, in the absence of SOCS2, DSS treatment resulted in less severe changes to the trabecular bone architecture. This underscores the importance of *Socs2* ablation in protecting against bone loss in DSS-induced IBD. Complete protection of the skeleton was not observed, however, and this is likely due to other cellular mechanisms, including increased osteoclastic resorption, direct (non-GH-mediated) effects of proinflammatory cytokines on osteoblast differentiation/function and diminished GH signaling through elevated *Socs3* levels. The effects of ablating both *Socs2* and *Socs3* in the DSS model would be of interest as it may offer enhanced protection to the skeleton. Previous studies have shown that the removal of GH signaling deletes the *Socs2*^−/−^ phenotype, including the normalization of body weight and bone length (Greenhalgh et al., 2002, 2005). Therefore, it is likely that mice in which both *Socs2* and GH signaling are silenced have no protection from DSS-induced bone loss. The gathering of such evidence would provide functional evidence for GH's pivotal role in protecting bone health in this animal model of IBD. Furthermore, it is also worth noting that reduced expression of SOCS2 has been associated with increased cancer risk, which may be a consequence of increased GH signaling (Hendriksen et al., 2006). Therefore, if GH treatment or novel therapies to target the SOCS2 protein are to be considered as bone-protective agents in children and adults with IBD, then the potential cancer risk has to be carefully considered.

Histological scoring in the present study was carried out using a validated scoring scheme, allowing an in-depth assessment of mucosal integrity (Dieleman et al., 1998; Williams et al., 2001). Acute and chronic colitis are characterized by distinct pathological changes to the colon. Acute inflammation is associated with an influx of neutrophils into the lamina propria and, in some cases, epithelial degeneration. Chronic inflammation, on the other hand, is associated with mononuclear leukocyte infiltration, crypt architectural disarray and crypt regeneration (Melgar et al., 2005; Perše and Cerar, 2012). In this study, histological analysis revealed signs of both acute and chronic inflammation in the colons of all DSS-treated mice, suggesting that the current experimental design was sufficient to induce chronic inflammation. Previous reports have shown that acute inflammation can progress to chronic in C57BL/6 mice following a single treatment of DSS (Melgar et al., 2005). Importantly, little difference was observed between the pathology scores of colons from DSS-treated WT and *Socs2^−/−^* mice. This pathological assessment suggests that the absence of SOCS2 does not appear to protect against the deterioration of mucosal integrity in DSS experimental colitis and therefore the improved bone health noted in the DSS-treated *Socs2*-deficient mice is unlikely to be solely attributed to improved disease status. However, it is recognized that further studies to quantify cell apoptosis, proliferation and stem cell number within the colon would allow us to make a more definitive judgement on colon health in the DSS-treated WT and *Socs2*-deficient mice.

Intriguingly, there have been reports that increased GH activity promotes mucosal repair during IBD-associated inflammation. A small clinical trial of patients with active CD reported that therapy with rhGH improved the CD activity index and decreased the need for other medication (Slonim et al., 2000). Previous studies have also reported that, in mice with a mutated IL-6 receptor subunit beta (gp130 receptor; gp130Y757F mice), there is protection to the colon from the damaging effects of DSS (Bollrath et al., 2009). It is possible that, in the gp130Y757F mice, in which SOCS3 cannot inhibit JAK/STAT signaling, there is a protective pathway(s) involving increased STAT3 activation that may not be active in SOCS2 deficiency*.*

Furthermore, GH transgenic mice display a similar extent of colon pathology during the onset of inflammation compared to WT mice, but show improved mucosal repair over an extended time period (Williams et al., 2001). Increased mucosal repair in the GH transgenic mice may be a result of increased systemic IGF-1, which has been reported to partially attenuate colonic damage in the DSS rat model (Mathews et al., 1988; Howarth et al., 1998). Although we noted subtle improvements in the crypt damage/regeneration score of DSS-treated *Socs2^−/−^* mice compared with DSS-treated WT mice, this is unlikely to be related to the systemic IGF-1 status of these mice, which was found to be normal. However, it must be noted that gut IGF-1 levels were not measured in these mice. Therefore, the possibility of local upregulation of IGF-1 in response to unregulated GH signaling in *Socs2^−/−^* mice cannot be ruled out. Despite the slight improvement in the crypt damage/regeneration score of DSS-treated *Socs2^−/−^* mice, their total pathology score was similar to DSS-treated WT mice. It is, however, prudent to note that, in our studies, systemic IGF-1 levels were measured at the end of the experiment, when the DSS-treated animals presented with severe inflammation of the colon, but had recovered their body weight. Previous studies have shown decreased systemic IGF-1 levels with active disease that returned to normal with recovery (Harris et al., 2009). Further studies are required to understand fully the role of systemic IGF-1 in the skeletal response to experimental IBD. In keeping with previous research, systemic IGF-1 levels in WT and *Socs2^−/−^* control animals were comparable, further confirming the importance of the direct anabolic effects of GH on bone (Metcalf et al., 2000; Lorentzon et al., 2005; MacRae et al., 2009; Dobie et al., 2014; Dobie et al., 2015).

The negative effects of increased *Socs2* expression in inflammatory conditions may not be restricted to the skeleton. In rodent models of chronic kidney disease there is increased *Socs2* gene expression in liver and muscle, which may contribute to impaired phosphorylation and nuclear translocation of GH-activated STAT proteins and the development of GH resistance (Schaefer et al., 2001; Sun et al., 2004; Mak and Cheung, 2007; Cheung et al., 2008). Intervention strategies to reduce uremic cachexia are associated with amelioration of the uremia-associated increase in *Socs2* expression (Cheung et al., 2008). Recent data have also shown that *Socs2* deletion protects against streptozotocin-induced type 1 diabetes in adult male mice, possibly through increased β-cell hypersensitivity to GH (Alkharusi et al., 2016). It is therefore likely that SOCS2 signaling represents a generic critical pathway through which proinflammatory cytokines alter both GH/IGF-1 signaling and cellular function (Ahmed and Farquharson, 2010; Farquharson and Ahmed, 2013).

In conclusion, these studies suggest that the absence of SOCS2 is protective against bone loss typical of IBD and are consistent with the premise that increased osteoblast SOCS2 expression represents a critical mediator through which proinflammatory cytokines inhibit GH signaling and decrease osteoblast function and bone accrual. This study also provides an improved understanding of the relative effects of GH/IGF-1 on bone health in experimental colitis and is consistent with data reporting the beneficial effects of GH on bone in conditions such as juvenile idiopathic arthritis (Bechtold et al., 2009). This accumulation of information is essential before these drugs are explored as bone-protective agents in children and adults with IBD.

## MATERIALS AND METHODS

### Mice

It is recognized that different genetic strains of mice respond differently to DSS (Melgar et al., 2005; Perše and Cerar, 2012). Therefore, we backcrossed our original *Socs2*-knockout (KO) mice (76.0% C57BL/6) (MacRae et al., 2009) with pure C57BL/6 mice a further six times to establish our *Socs2*-KO mice and littermate WT controls on a pure (>99.0%) C57BL/6 background strain. These mice were used in all studies reported. For genotyping, ear-biopsied DNA was analyzed by PCR for *Socs2* (WT) or the neocassette (*Socs2*^−/−^) using the following primer sequences: SOCS2-Forward (5′-3′) TGTTTGACTGAGCTCGCGC, Reverse (5′-3′) CAACTTTAGTGTCTTGGATCT and Neo-Forward (5′-3′) ACCCTGCACACTCTCGTTTTG, Reverse (5′-3′) CCTCGACTAAACACATGTAAAGC. Primers were obtained from Eurofins MWG Operon, London, UK. All animal experiments were approved by Roslin Institute's Animal Users Committee, and the animals were maintained in accordance with Home Office (UK) guidelines for the care and use of laboratory animals.

### Establishment of the acute DSS-induced colitis model

Male WT and *Socs2*-KO mice (six mice per group), 8-9 weeks of age, received 3.0% DSS (molecular mass ∼40,000 kD; Sigma Aldrich, UK) in their drinking water (tap water). They were given DSS-treated water *ad libitum* for 4 days, following which they received normal tap water for a 14-day recovery period. Control (non-DSS-treated) male WT and *Socs2*-KO mice (six mice per group) were offered normal tap water for the duration of the study. The health status of the DSS-treated mice was scored daily, with particular attention paid to their coat condition, mobility, presence of blood in stools and eye clarity. Body weights of all mice were recorded daily. To establish the weight loss that was due to inflammation and not lowered food intake, the mice were pair-fed. The quantity of food consumed daily (fed *ad libitum*) by the DSS-treated mice was weighed and then provided to control animals (who received no DSS) the following day (Ballinger et al., 2000). All mice were housed individually to allow accurate measurement of food and water intake and health status. At 18 days after the initiation of the studies, the experiment was stopped, blood collected for serum analysis, and the long bones and the colon dissected.

### Colon histology

The colon was dissected from WT and *Socs2^−/−^* mice±DSS treatment and fixed and stored in 4% paraformaldehyde. Each colon was divided into five transverse segments, including proximal to distal portions. Tissue processing, wax embedding, sectioning (5 µm thick) and H&E staining were done following routine procedures. Colon pathology was graded blind on sections from all five segments of each mouse using an established histological grading scheme (Dieleman et al., 1998). Segments of colon were assessed separately for inflammation. Scores from all five segments were averaged to provide an overall pathology score. Colon diameters were measured on the H&E sections. Using image analysis software, the diameter of each transverse segment was measured twice and averaged.

### Micro-computed tomography

To evaluate trabecular architecture and cortical bone geometry of the tibia from control and DSS-treated mice, we used a µCT system (SKYscan 1172 X-ray microtomograph, Bruker Corporation, Kontich, Belgium) as described previously (Dobie et al., 2014). In brief, high-resolution scans with an isotropic voxel size of 5 µm (trabecular bone) or 10 µm (cortical bone) were acquired (60 kV, 0.5 mm aluminium filter, 0.6° rotation angle). Two images were averaged at each rotation angle. Scan reconstruction was done using NRecon software (Bruker). A 1 mm section of the metaphysis was taken for the analysis of trabecular bone, using the base of the growth plate as a standard reference point. A 500 µm section of the mid-shaft was taken for the analysis of cortical bone, using the articulation with the fibula as a standard reference point. CTAn software (Bruker) was used to analyze the appropriate parameters (Bouxsein et al., 2010).

### Serum IGF-1 ELISA

Blood obtained by cardiac puncture was stored in serum tubes (Greiner Bio-One, Gloucestershire, UK) on ice for over 30 min to allow for clotting. Following centrifugation for 10 min at 1000 ***g***, supernatant was removed, aliquoted, and stored at −80°C. IGF-1 levels were assessed by ELISA (Quantikine, R&D Systems, Minneapolis, USA) according to the manufacturer's instructions.

### RNA extraction and RT-qPCR analysis

Left femur were dissected from WT and *Socs2^−/−^* mice±DSS treatment. The femurs had both epiphyses removed and the marrow was spun out by centrifugation and discarded. The bone samples were snap frozen in liquid nitrogen and stored at −80°C. Bone samples were ground using a mortar and pestle, and homogenized by a hand-held homogenizer in QIAzol Lysis Reagent. RNA was extracted using an RNeasy Lipid Tissue Kit (Qiagen Ltd, Manchester, UK) following the manufacturer's protocol. RNA content was measured by absorbance at 260 nm, and quality by 260/280 ratios. RNA was stored at −80°C. Reverse transcription (RT) was carried out as described previously (Newton et al., 2012). RT-qPCR was performed using the SYBR Green [Roche detection method on a Stratagene Mx3000P real-time qPCR machine with MxPro software (Stratagene, Santa Clara, CA, USA)]. Relative gene expression was calculated using the ΔΔCt method (Livak and Schmittgen, 2001). Each cDNA sample was normalized to the housekeeping gene *Gapdh* (Primer Design, Southampton, UK). Reactions were performed with gene-of-interest primers (5′-3′): *Socs1* (Forward TCCGATTACCGGCGCATCACG; Reverse CTCCAGCAGCTCGAAAAGGCA), *Socs2* (Forward TGGCTGCTCAAGATCAAATG; Reverse TGTCCTCCTGGAAATGGAAG) and *Socs3* (Forward GAGTACCCCCAAGAGAGCTTACTA; Reverse CTCCTTAAAGTGGAGCATCATACTG) (MWG Eurofins).

### Statistical analysis

All measurements and analyses were done blinded to the researcher. All statistical analysis was completed using GraphPad Prism. Final measurements, IGF-1 ELISA and histology scoring data were analyzed using a two-tailed unpaired *t*-test with Welch's correction (equal standard deviations not assumed) or suitable nonparametric test (Mann–Whitney) if the data were not normally distributed. Owing to the small sample size, transcript and μCT data were analyzed using a nonparametric test (Mann–Whitney). Data are presented as means±s.e.m.

## Supplementary Material

Supplementary information
