# Supplementary material for: Suppressor of cytokine signaling 2 (Socs2) deletion protects bone health of mice with DSS-induced inflammatory bowel disease
Source: Dis Model Mech. 2018 Jan 1;11(1):dmm028456. doi: 10.1242/dmm.028456 (PMC5818069; doi:10.1242/dmm.028456)
Supplement: Supplementary information [file dmm-11-028456-s1.pdf]

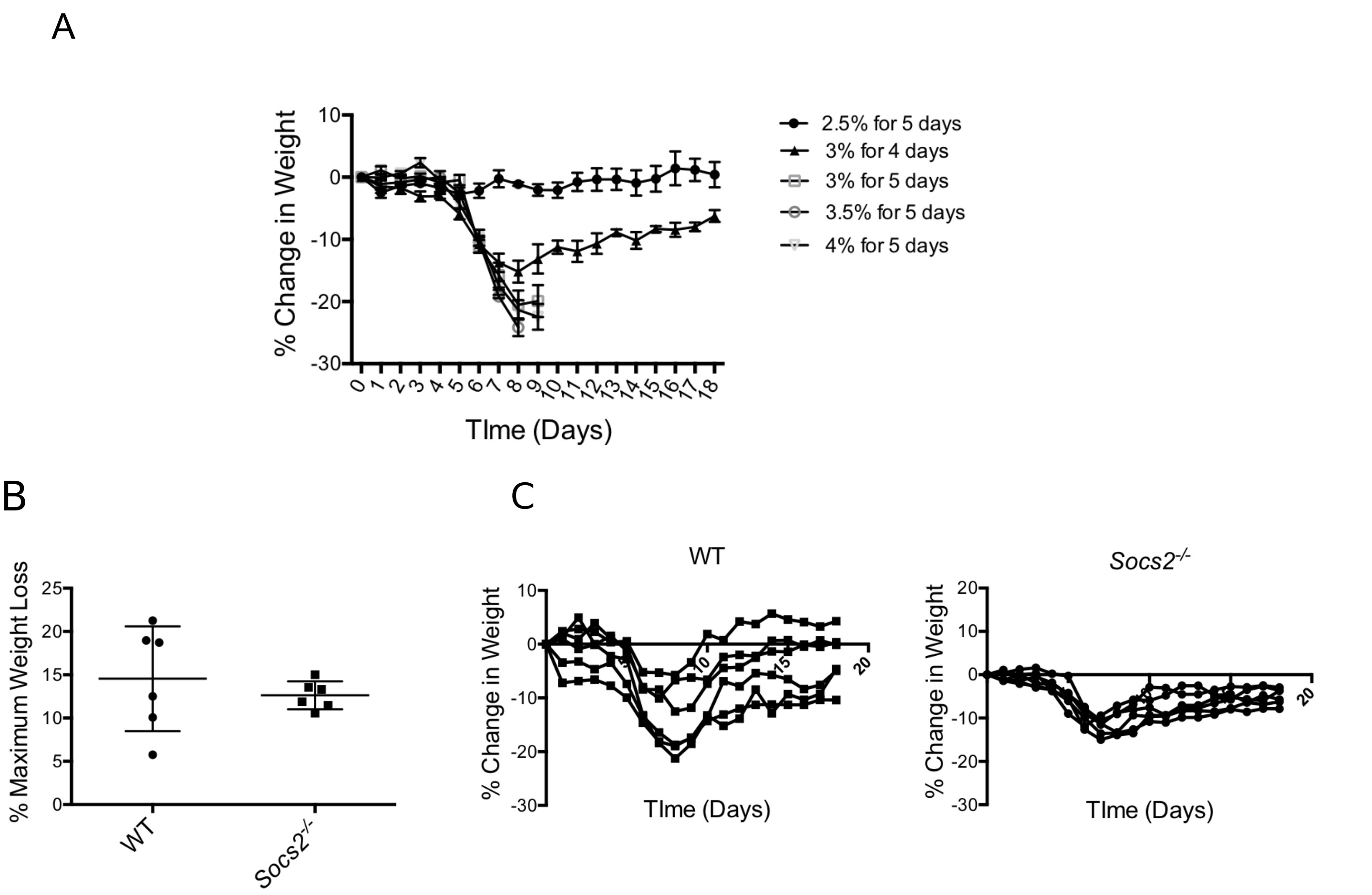

Supplementary Figure 1. **DSS concentration and time dependent changes in body-weight. A.** Body weight change of WT mice subjected to varying doses and treatment times of DSS. **B.** Maximum weight loss observed by WT and *Socs2*$^{-/-}$ during DSS experiment. Data presented as mean± SEM (n=6). Data non-significant by unpaired t-test. **C.** Individual weight change observed in WT and *Socs2*$^{-/-}$ mice treated with 3% DSS.

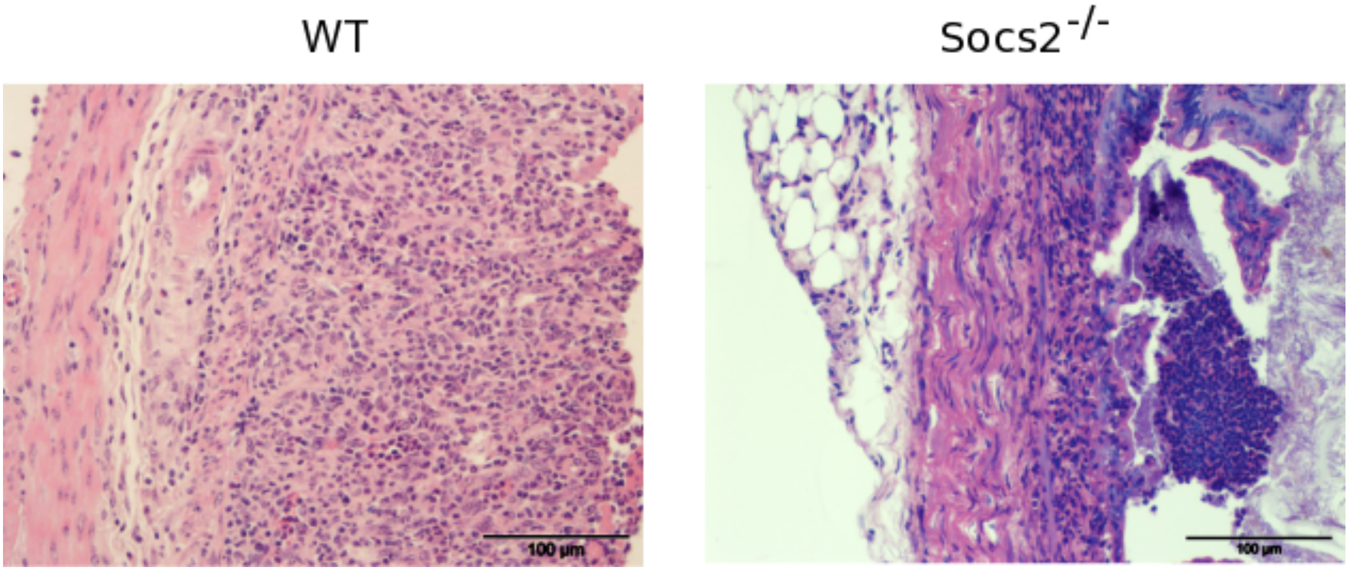

Supplementary Figure 2. **Effects of DSS on mucosal integrity of WT and *Socs2*$^{-/-}$ mice.** Representative image from WT and *Socs2*$^{-/-}$ mice treated with DSS. Images show epithelial degeneration, crypt loss, high levels of mononuclear leukocytes and transmural inflammation.
